# Effect of Microresistor Topology on the Sensing Characteristics of MoS_2_-Based Chemoresistive Cortisol Sensors

**DOI:** 10.3390/s26020551

**Published:** 2026-01-14

**Authors:** Mariya Aleksandrova, Rade Tomov, Boriana Tzaneva, Ivo Iliev

**Affiliations:** 1 Department of Microelectronics, Technical University of Sofia, 1756 Sofia, Bulgaria; rtomov@tu-sofia.bg; 2 Department of Chemistry, Technical University of Sofia, 1756 Sofia, Bulgaria; borianatz@tu-sofia.bg; 3 Department of Electronics, Technical University of Sofia, 1756 Sofia, Bulgaria; izi@tu-sofia.bg

**Keywords:** microresistor topology, MoS_2_ nanocoatings, cortisol sensor, wearable sensors

## Abstract

This study investigates the impact of microresistor topology on the sensing characteristics of MoS_2_-based chemoresistive cortisol sensors. It is done to address the critical need for robust, non-invasive cortisol monitoring in wearable applications, where mechanical stability under strain is paramount, and to explore underexplored topological effects on sensor performance. The research is conducted by fabricating MoS_2_-based meander structures on flexible PDMS substrates, featuring various microresistor designs, including long-shoulder and short-shoulder topologies, both with and without integrated mechanical ribs. Sensor performance is evaluated in resistance change mode across a range of cortisol concentrations (2.5 to 500 ng/mL) and subjected to significant mechanical bending stress. Electrical parameters such as contact resistance and parasitic capacitance, as well as temperature dependence, are also analyzed. The results demonstrate that the incorporation of ribs significantly enhances the mechanical stability and preserves the reliable sensing function of the long-shoulder topology under bending stress, improving sensitivity from 0.9 kΩ/ng/mL (without ribs) to 130.6 kΩ/ng/mL (with ribs) after bending. While temperature influences baseline resistance and response magnitude consistent with MoS_2_ semiconductor properties and aptamer binding kinetics, the short-shoulder design, even with ribs, showed less optimal performance. The primary advantage of the proposed device lies in its enhanced mechanical reliability under continuous strain, crucial for wearable electronics, alongside a simpler design compared to complex microfluidic or optical systems, thus enabling lower manufacturing costs and easier mass production.

## 1. Introduction

Cortisol is an important glucocorticoid hormone, involved in the regulation of many aspects of metabolism, stress and immune response, monitoring of reproductive cycles, diagnosis and treatment of hormonal disorders, and optimization of athletic training regimes. It is therefore important to monitor its concentrations in a non-invasive way for the betterment of public health [1]. Non-invasive methods for cortisol measurement, particularly through sweat analysis, have gained attention due to their potential for real-time monitoring [2]. The electrochemical cortisol detector has been the most common approach for wearable sweat sensors due to its potential for miniaturization. These sensors work by converting chemical signals in the sweat into measurable electrical signals (e.g., changes in impedance) [3]. They are currently more mature for clinical applications, offering also robust performance and superior stability over standard antibodies [4]. Colorimetric sensors rely on chemical reactions that produce a color or fluorescence change when cortisol is present. The color change can often be analyzed visually or with an external device like a smartphone for semi-quantitative results [5]. The biosensors are integrated into various wearable platforms such as skin patches, tattoo-based sensors, smart textiles (e.g., integrated into headbands or wristbands), and smartwatches [6,7,8,9].

Molybdenum disulfide (MoS_2_) has been studied for its potential in cortisol sensing [10]. It is noted for its unique electrical, optical, mechanical properties, high surface area, and tunable bandgap, which make it an ideal material for development of highly sensitive and selective sensors for various applications, including medical diagnostics, environmental monitoring [11], cancers cell detection [12] and non-enzymatic biosensors that can detect hormonal changes in biological fluids, such as human sweat [13]. Its atomically thin, flexible nature makes MoS_2_ an ideal material for developing miniature, portable, and wearable sensing devices for real-time, continuous monitoring. It also exhibits good biocompatibility, minimizing irritation when integrated into wearable or implantable biomedical devices. MoS_2_-based platforms have achieved exceptionally low limits of detection (LOD) for cortisol, reaching nanogram per milliliter range, which is clinically relevant (8.16 to 141.7 ng/mL in sweat) [14]. Sensors incorporating MoS_2_ have demonstrated fast response times within a few minutes for continuous monitoring, demonstrating a significant improvement over traditional lab-based methods like Enzyme-Linked Immunosorbent Assay (ELISA), which can take hours. The intrinsic mechanical flexibility and strength of MoS_2_ nanosheets allow for integration into durable, stretchable, fabric-based, and unobtrusive wearable patches that can withstand strain without performance degradation [15,16]. A widely utilized and effective strategy for creating highly sensitive MoS_2_ aptasensors involves the use of biotin-labeled aptamers in conjunction with the high surface area and electronic properties of MoS_2_ nanosheets. The sensor functions based on a conformation change mechanism. In the absence of cortisol, the aptamer is in a specific conformation (often a random coil or G-quadruplex-like structure). When cortisol binds, the aptamer undergoes a conformational change, folding into a target-bound structure. This alteration in aptamer structure and its associated surface charge generates an electrical signal (e.g., current/voltage change in a resistive sensor), which is proportional to the cortisol concentration. MoS_2_ nanosheets act as excellent signal transducers, using their high surface-to-volume ratio and direct electronic interaction with the aptamer layer to produce clearly measurable conductivity changes on the contact pads.

In this research, we explore the potential of the chemoresistive response principle as a viable sensing mechanism, focusing on how it induces a quantifiable shift in electrical resistance or conductance. The operation of these biosensors primarily relies on the chemoresistive response principle, where exposure to target analytes results in measurable alterations in the conductance of the sensing material [17]. A chemoresistive approach is selected over electrochemical or optical methods primarily due to potential advantages in simplicity of fabrication, long-term operational stability, and the ability to tailor their characteristic response according to the microresistors’ topology (long- and short-shoulder configurations). The fundamental principle of measuring resistance change is straightforward to integrate into miniaturized, flexible, and wearable platforms. While previous studies have explored the electrical properties of MoS_2_ in sensor applications [18], the specific effects of microresistor topologies on the performance of MoS_2_-based cortisol sensors remain underexplored.

To design effective chemoresistors, a variety of topologies can be proposed. The most basic classification relates to the physical arrangement of the sensing material and the electrodes. Planar type is a common configuration where a thin or thick film of sensing material is deposited onto a flat insulating substrate (like ceramic or silicon) containing pre-deposited interdigitated electrodes [19]. Micromachined (on-chip) chemoresistive devices are fabricated using top-down microfabrication processes directly on a chip substrate, often including an integrated micro-heater on the backside to control the operating temperature [20]. Comparing the mechanical stability of a chemiresistive sensor using MoS2 films on a patterned gold-coated PDMS (polydimethylsiloxane) substrate to traditional planar-type and micromachined (on-chip) sensors reveals significant differences in design philosophy and mechanical performance—a shift from high rigidity to exceptional flexibility and stretchability [21]. The basic features of the main cortisol sensor design are summarized in Table 1.

In this study, specific designs for the microresistors were proposed, incorporating additional ribs for enhancing the mechanical stability on bendable substrates and reducing errors caused by deformation. Thus, the general aim of the research is to investigate how sensing element topologies and reinforcing fragments impact performance to address the mechanical fragility of traditional cortisol sensors in wearable applications. To the author’s knowledge, this is the first investigation into how the presence, size, and configuration of these ribs affect the sensor’s sensitivity. The length and the pitch of the microresistor lines were varied, and different cortisol concentrations were measured in resistance change mode. The results show that the incorporation of ribs in the chemoresistive design has a negligible effect on the resistance variation with respect to cortisol concentration for the long-shoulder design. At the same time, this design demonstrates improved stability against bending compared to the ribless microresistor and the short-shoulder topology with ribs. Additionally, it was found that the sensor performance in the short-shoulder configuration is impaired. Thermal stability of the sensors was also investigated to provide information on the sensor behavior when contacting the human body. The results provide valuable insights into optimal design strategies for chemoresistive cortisol sensors, thereby advancing the field of non-invasive hormone monitoring and contributing to improved public health outcomes.

## 2. Materials and Methods

The insertion of intermediate ribs between the “shoulders” (the straight, parallel sections) of meander wires enhances the resistance stability of a strain resistor by employing a strategy of mechanical reinforcement and controlled strain redistribution. This design methodology primarily addresses issues of delamination, material fatigue, and non-uniform deformation under repeated mechanical stress [22]. The primary role of the ribs is to act as structural supports that limit excessive or unpredictable mechanical deformation of the sensor structure during strain cycles. By integrating these features, especially in flexible or textile-based sensors, the ribs help maintain the overall geometry of the sensor when it is bent, stretched, or compressed. This physical confinement prevents the meander wires from buckling or moving excessively out of plane, which would otherwise lead to unstable resistance readings, which is not due to the cortisol concentration change, thus preventing measurement error when wearing.

The initial resistance R_0_ of the meander-shaped conductive path is determined by its material resistivity *ρ*, total length *L*, and cross-sectional area *A* = *w* × *t*, where *w* is the width and *t* is the thickness (Equation (1)):

(1) $$R_{0}=\rho \frac{L}{w\times t}$$

Gauge factor *GF* links the fractional change in resistance *ΔR/R*_0_ to the applied mechanical strain *ε* (Equation (2)).

ΔR/R_0_ = GF × ε (2)

For a simple metal strain gauge, *GF* accepts a value typically around 2. For advanced nanomaterials, such as MoS_2_, the gauge factor is much higher (more than 100) due to changes in resistivity with strain (the piezoresistive effect), not just dimensional changes [23].

In the presented case, it should be taken into account that the resistivity is complex and formed by the electrode film resistivity together with the sensitive film resistivity.

The dimensions of the meander pattern are crucial design parameters involving total length *L*, determined by the number of turns and the length of each straight segment (shoulder) within the active gauge length; grid line width w and spacing (pitch); and gauge length *L_G_*, which is the overall length of the area covered by the meander. Longer *L* increases *R*_0_ but does not inherently increase *GF* in an ideal linear strain field. Narrower line widths can lead to a slightly better *GF* in certain material systems and help localize strain measurement. Spacing must be sufficient to prevent shorting during maximum expected deformation.

Specific analytical formulas to directly calculate optimal rib dimensions (height, width, placement) for a desired *GF* are not generally available as they depend heavily on the specific sensor system, substrate, and geometry. Instead, the methodology relies on the Theory of Elasticity equations used within FEA calculations to model stress and strain distribution. The design of the ribs aims to modify the local strain *ε_local_* experienced by the meander compared to the nominal applied strain *ε_applied_*, effectively attenuating the strain amplification factor (*SAF* < 1). The *SAF* is determined by the contrast in Young’s Modulus *E* between the soft substrate (or flexible part of the structure) and the rigid rib material.

**Figure 1:**
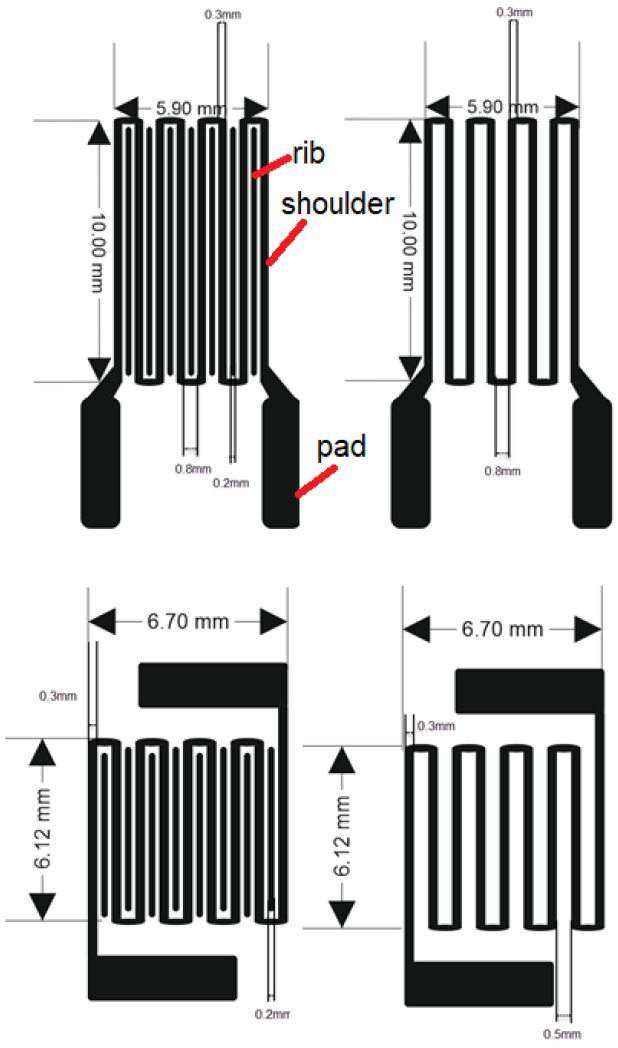
Chemoresistive cortisol sensor designs with different rib and shoulder geometry patterns.

Based on this methodology, the dimensions of the investigated topology were achieved as shown in Figure 1.

Gold was chosen as the electrode for its excellent conductivity and also its mechanical properties in thin film form. When a thin layer of gold is deposited on polydimethylsiloxane (PDMS) flexible substrate (Merck, Darmstadt, Germany), it spontaneously forms a network of nanoscale cracks. These micro-cracks can open and close reversibly under mechanical strain (bending/stretching) without catastrophic electrical failure, which provides reliable electrical performance under deformation [24]. The strong adhesion between Au and S atoms in MoS_2_ (2D Semiconductors, Scottsdale, Arizona, USA) also helps maintain structural integrity at the interface. Gold coating with a thickness of 450 nm was DC sputtered at a base pressure of 2.5 × 10^−2^ Torr and sputtering current of 40 mA. Conventional photolithographic patterning with wet chemical etching of gold in a potassium iodide non-aggressive solution was applied to create the desired geometries according to Figure 1. MoS_2_ thin films with a thickness of 600 nm were grown by ultrasonic spray coater Siansonic (Beijing, China) benchtop system from isopropyl alcohol solution with a viscosity of 2 cPs. The liquid feeding was 0.4 mL/min, the carrier gas pressure was 0.019 psi, the ultrasound generator’s power was 1.7 W, and the temperature of spraying was 80 °C. The optimal nozzle scan rate was found to be 6 mm/sec. An aptamer was then applied as the capture molecule to selectively bind with cortisol by spin-coating over MoS_2_ at 2500 rpm for 30 s and dried at 70 °C to stabilize the coating. Synthetic DNA aptamer to cortisol [CSS.1] (Biotin) produced by Antibodies (St. Louis, Missouri, USA) was used. Since the aptamer is delivered chemically modified with biotin, with thiol functional group termination at the 3′ end [25], it has a high propensity for chemisorption interactions. Therefore, it can be assumed that the immobilization of the aptamer occurs through a chemisorption of the thiol group at the S vacancy sites of MoS_2_. This allows the attachment of the aptamer to MoS_2_ through the spontaneous Mo-S bond [25,26].

Cortisol solutions based on Dimethylformamide solvent (DMF) with cortisol concentrations in the range from 2 ng/mL to 500 ng/mL were drop-casted by automatic micropipette. Four-terminal setup, or the Kelvin technique, was applied for the electrical characterization of the samples to ensure the highest possible accuracy and precision in resistance measurements by eliminating the effect of contact resistance. In addition, an impedance analyzer, Hioki IM3570 (Hioki, Nagano, Japan), was connected to measure the contact resistance and parasitic capacitance extracted from the impedance in a frequency range of 1 Hz to 200 kHz and bias voltage of 0.005 V. They were measured to track the quality of the electrical contact after bending.

**Figure 2:**
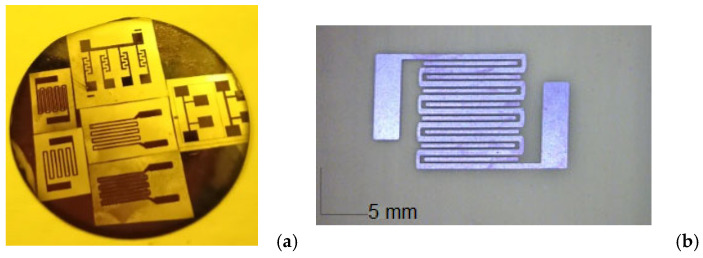
Fabricated chemoresistive references sensing structures: (**a**) all topologies on a standard rigid silicon wafer; (**b**) a microscopic view of the short-shoulder meander resistor with additional ribs.

The different geometries of the chemoresistors were explored at bending with an intensity of 100 g-force/cm^2^, frequency of 17 Hz, and 1000 repeating bending cycles of tension and compression. The bending cycles were executed according to a methodology developed specifically for wearable biosensors and reported elsewhere [27]. The low-frequency range of the applied force aims to imitate human limbs folding. These bending parameters were specifically chosen to realistically emulate the repetitive mechanical stresses experienced by wearable sensors adhered to skin over major joints, such as the wrist or elbow, during activities like walking, grasping, or typing, where continuous limb motion subjects the device to dynamic force cycles and cumulative strain. At the same time, they have to serve as an accelerated aging (stress) test; therefore, they must be more intensive then the typical values for normal activity. In microelectronics, a stress test pushes devices beyond normal operating limits to find failure points, assess reliability, and ensure they survive real-world conditions, identifying design flaws and determining lifespan, often involving accelerated aging for early failure detection. Different temperatures from room temperature to 40 °C which are relevant as operational temperatures taking into account that the normal human temperature is average 36.7 °C, were set by a lab-made temperature regulator with a Peltier heater. All measurements were conducted on pairs with ribbed and ribless microresistive structures. They were first conducted on microresistors fabricated on a rigid substrate (Si wafer) to validate the concept for the effect of the rib presence and absence on the initial resistance (Figure 2a) and compare with reference test resistive structures. Then the technology flow was transferred to the flexible substrate (Figure 2b). Then the technology flow was transferred to the flexible substrate (Figure 2b). In this way, the electrical performance of the sensing material and structure was isolated from potential mechanical artifacts introduced by a flexible substrate and a reliable baseline was established. All measured responses are representative of consistent results obtained from four independently fabricated devices of each type.

## 3. Results and Discussion

**Figure 3:**
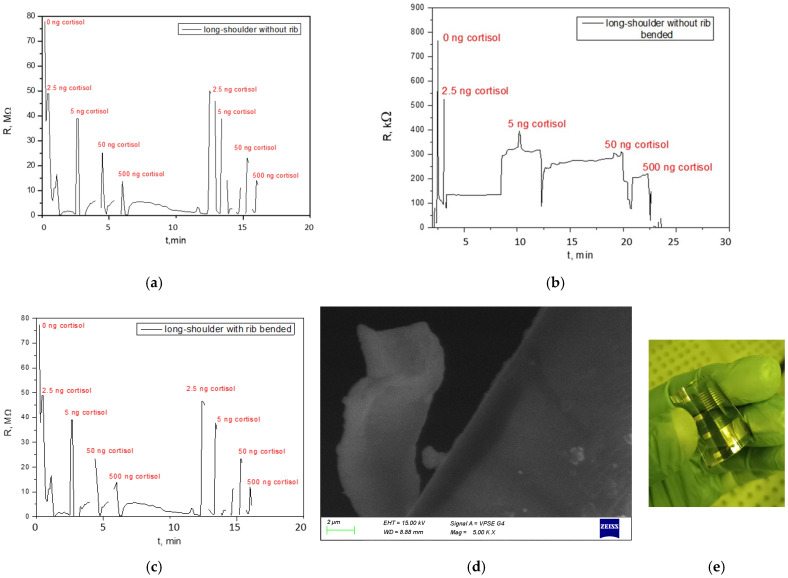
Dynamic response of a resistive sensor exposed to different concentrations of cortisol over time at room temperature: (**a**) for long-shoulder topology without rib before bending; (**b**) for long-shoulder topology without rib after bending; (**c**) for long-shoulder topology with rib and after bending; (**d**) SEM image of the bended structure without rib; (**e**) photo of the two fabricated sensor structures on a common substrate.

Figure 3a displays the resistance (*R* in MΩ) over time (t in min) at different cortisol concentrations and room temperature of a MoS_2_-based cortisol sensor with long-shoulder topology without additional ribs. It shows distinct peaks and changes in resistance when different concentrations of cortisol (0, 2.5, 5, 50, 500 ng/mL) are applied, indicating a functional sensor. The second pass with the same concentrations reveals similar peak intensity (resistance change magnitude), which is an indication of repeatable response. The observed repeatability demonstrates short-term reusability, crucial for continuous monitoring in wearable contexts. The sensor exhibits clear and relatively sharp resistance changes upon exposure to varying cortisol concentrations. Higher concentrations appear to induce more significant or distinct resistance shifts. This serves as a baseline showing the sensor’s performance in an ideal, unstrained state.

This MoS_2_-based chemoresistive sensors operate on the principle of resistive-induced field-effect modulation. Upon cortisol binding, the aptamer undergoes a specific conformational change, altering its spatial arrangement and charge distribution near the MoS_2_ surface. This change in the local electrostatic environment effectively modulates the carrier concentration within the MoS_2_ nanosheets. As the measurements demonstrate, the sensors exhibit a decrease in resistance upon cortisol binding. This indicates an increase in electron concentration within the MoS_2_ conduction channel, consistent with the behavior of an n-type semiconductor. This phenomenon suggests that the aptamer-cortisol complex, in its bound conformation, either acts as an electron donor to the MoS_2_ or, more likely, reconfigures its charge distribution in a way that effectively reduces a prior electron-depleting effect, or creates a more positive local environment that attracts and accumulates electrons in the n-type MoS_2_ pathways. The precise nature of this charge transfer or similarity to gating modulation is complex and influenced by the specific aptamer structure and its interaction with the MoS_2_ surface. We assume that the aptamer-cortisol interaction does not have a direct relation to the topology; therefore, the detailed study of the stated hypothesis will be presented in subsequent studies.

Figure 3b shows resistance (*R* in kΩ) over time for the same sensor (long-shoulder, no ribs), but after being subjected to bending stress. In contrast to the results, presented in Figure 3a, the sensor’s response after bending is severely degraded. The peaks are less distinct, highly noisy, and the baseline resistance appears more variable. The ability to clearly differentiate between cortisol concentrations is significantly compromised. Without structural support of the ribs, bending causes brittle fracture, delamination, or nanocracking of the films. Lattice strain and micro-scale discontinuities increase the distance between conductive domains, forcing current through fewer or more paths, which leads to unstable resistance and electrical noise. The mechanical delamination of the MoS_2_ film from the substrate or internal bond-breaking due to strain decouples the biological sensing layer (aptamer) from the transducer (MoS_2_), resulting in a complete loss of signal reliability. In addition, tensile strain decreases the bandgap of monolayer MoS_2_, while compressive strain can either increase or decrease it depending on its magnitude [28]. Thus, without reinforcement ribs to stabilize the MoS_2_ response after bending, it cannot be unambiguously predicted if the resistance change is due to the cortisol concentration change or the strain-induced bandgap change. The mechanical failures disrupting the electrical pathways within the MoS_2_ sensing material lead to unstable resistance readings, electrical artifacts, and ultimately, a loss of reliable sensing capability. This was confirmed by the SEM image, presented in Figure 3d, showing a piece of the MoS_2_ coating delaminated from the flexible substrate after bending. Figure 3c presents the resistance (*R* in MΩ) over time for a sensor of the same long-shoulder topology, but now with integrated ribs, after being subjected to the same bending stress as the sensor from Figure 3b. The sensor’s response is significantly improved compared to the ribless sensor after bending. While not as sharp as the initially unbent, ribless sensor (Figure 3a), the peaks are more clearly defined, and the overall signal is much less noisy. The sensor’s ability to respond to different cortisol concentrations is largely restored. This figure directly demonstrates the core finding related to the effectiveness of incorporating ribs for enhanced mechanical stability. The ribs act as structural supports that limit excessive or unpredictable mechanical deformation. By preventing the meander wires from moving out of plane, they mitigate the mechanical degradation (micro-cracks, delamination) that fails the ribless sensor. A possible reason is effective strengthening of the flexible substrate locally, distributing mechanical stress more uniformly, and preventing critical strain concentrations that lead to crack initiation and propagation within the MoS_2_ layer. This maintenance of the sensor’s structural integrity translates to more stable electrical pathways and a more reliable chemoresistive response to cortisol, even under strain. Figure 3e shows a photo of the fabricated long-shoulder cortisol sensors with and without ribs on a common substrate.

**Figure 4:**
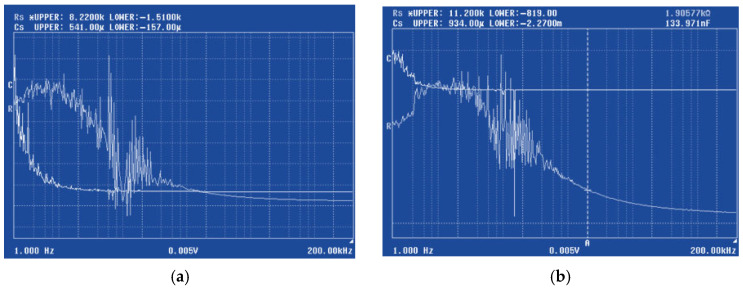
Contact resistance and capacitance after bending, measured for the long-shoulder topology: (**a**) with ribs; (**b**) without ribs.

Figure 4a,b show contact resistance (*R_s_*) and parasitic capacitance (*C_s_*) measurements, respectively, taken after bending for long-shoulder topology sensors. These electrical parameters are crucial for tracking the quality of the electrical contact after mechanical stress.

**Figure 5:**
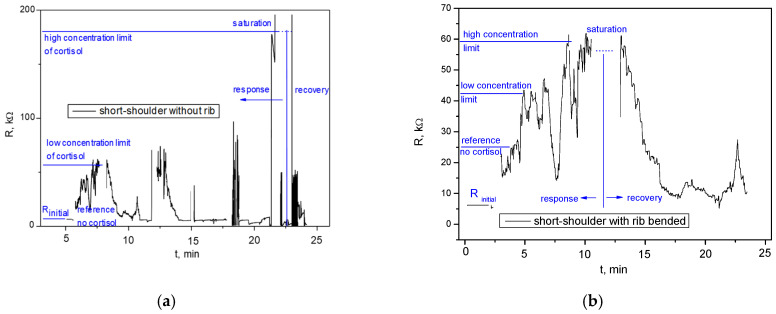

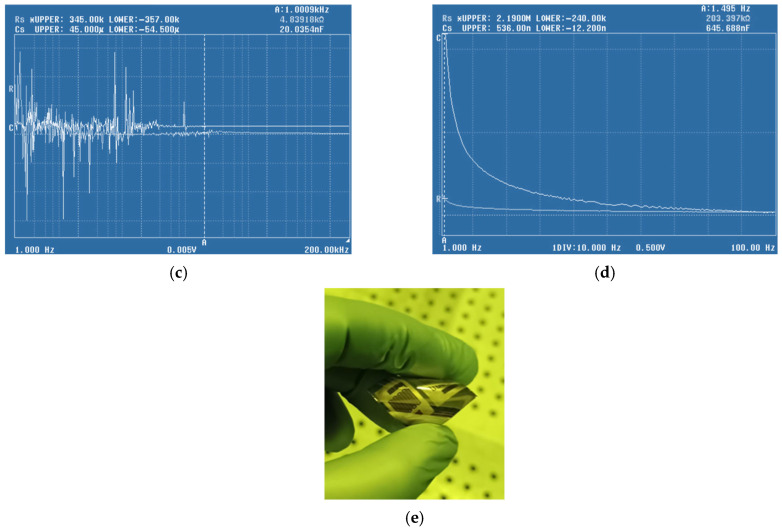
Dynamic response of a resistive sensor exposed to different concentrations of cortisol over time at room temperature: (**a**) for short-shoulder topology without rib after bending; (**b**) for short-shoulder topology with rib after bending; (**c**) contact resistance and capacitance, measured for the short-shoulder topology with ribs after bending and (**d**) without ribs after bending; (**e**) photo of the two fabricated sensor structures on a common substrate.

The recorded range in Figure 4a was for *R_s_* between 1.51 kΩ and 8.22 kΩ, and for *C_s_* between 157.00 μF and 541.00 μF. The recorded range in Figure 4b was for *R_s_* between 819 Ω and 11.2 kΩ and for *C_s_* between 2.27 mF and 934 μF. When comparing the ranges shown in 4b (without ribs) to 4a (with ribs), the ribless sensor shows a wider variation in both series resistance and capacitance, indicating greater electrical instability and variability in the ribless sensor after bending. It suggests that mechanical stress (bending) introduces more significant and unpredictable changes in the electrical connectivity between the sensing material and electrodes, primarily through interface delamination at the contact regions and micro-cracking within the MoS_2_ film, which directly impacts the effective contact area and current distribution. This directly supports the visual degradation seen in Figure 3b,d. The ribs, by providing structural integrity, help maintain more stable and consistent electrical contacts, leading to a narrower and potentially lower range of resistance fluctuations. Parasitic capacitance is influenced by the physical geometry of the device, the dielectric properties of the materials, and the proximity of conductive elements. A wider range of capacitance in the ribless sensor after bending implies that the physical structure of the device is undergoing more significant and variable deformation, affecting the spatial relationships between conductors. The ribs, by preserving the sensor’s geometry under strain, help to stabilize these capacitive elements, leading to a more consistent capacitance profile. The graph in Figure 5a shows the resistance over time for a short-shoulder sensor without integrated ribs, after being subjected to bending stress. The signal is noisy and erratic. While approximate high concentration limit, low concentration limit, saturation, and recovery are indicated, the signal fluctuations are so significant that it is difficult to reliably distinguish these states or specific quantitative responses to cortisol concentrations. For the short-shoulder topology, the susceptibility to mechanical degradation appears to be particularly pronounced, leading to an almost complete loss of reliable sensing function. Figure 5b displays the resistance over time for a short-shoulder sensor with integrated ribs, after the same bending stress. Compared to Figure 5a, there is a marginal improvement, with some fluctuations still present, but the limits of detection, response and recovery time, and sensitivity are vaguely more discernible, though still quite noisy and not as clean as the ribbed long-shoulder (Figure 3c). The remaining noise indicates that the mechanical stabilization is not as effective as for the long-shoulder, or that the short-shoulder geometry is inherently more prone to mechanical noise. The recorded ranges for R_s_ and C_s_ are notably wide, indicating significant electrical variability. The short-shoulder with ribs exhibits a substantially lower contact resistance and parasitic capacitance values (Figure 5c), as compared to the device without ribs (Figure 5d). The quantitative data supports the qualitative observation from Figure 5b. This geometry, even with ribs, is less optimal for sensing sensitivity, indicating that the short-shoulder topology is inherently more challenging to stabilize electrically under strain, or that the ribs interact differently with its specific dimensions.

Based on the experimental data where the lowest concentration (2.5 ng/mL) consistently produced a distinct and measurable signal above noise (as seen in Figure 3a,c), the Limit of Detection (LOD) was defined as 2.5 ng/mL. This is the lowest concentration for which a repeatable response was observed, as well. The experiments were conducted up to 500 ng/mL, which represents the upper limit of the cortisol concentration range investigated in this study. At this concentration, the sensor still exhibited a distinguishable response, thus defining a relatively broad range of detection, which is one of the advantages of the proposed sensor.

**Figure 6:**
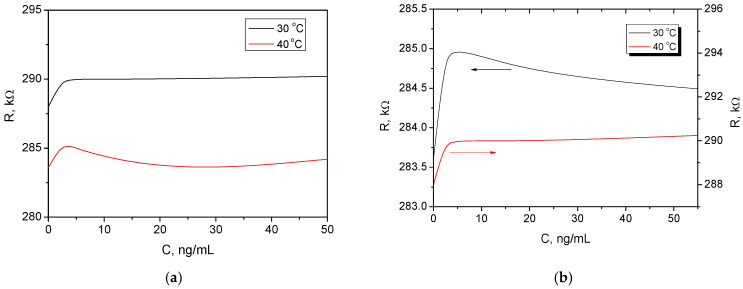
Temperature dependence of the chemoresistive response to cortisol at elevated temperatures for the long-shoulder topology: (**a**) before bending; (**b**) after bending.

Figure 6 illustrates the temperature dependence of the chemoresistive cortisol sensor (long-shoulder topology) both before and after bending, by showing the resistance (*R*) as a function of cortisol concentration (*C*) at two different temperatures (30 °C and 40 °C). Before bending (Figure 6a), the baseline resistance (at 0 ng/mL cortisol) is lower at 40 °C compared to 30 °C (e.g., ~283 kΩ at 40 °C vs. ~288 kΩ at 30 °C). The overall change in resistance (the sensor’s response magnitude to cortisol) appears slightly reduced at 40 °C compared to 30 °C, although the general trend is maintained. The overall resistance values in Figure 6b are higher after bending compared to before bending (e.g., baseline ~288 kΩ at 40 °C vs. ~283 kΩ in Figure 6a; ~290 kΩ at 30 °C vs. ~288 kΩ in Figure 6a. This increase in resistance is consistent with mechanical degradation or stress introduced by bending, which causes micro-cracks or delamination in this case, affecting electrical pathways. Similarly to 6a, the baseline resistance is lower at 40 °C than at 30 °C. The difference in response between 30 °C and 40 °C appears more pronounced after bending, with the 40 °C curve showing a lower overall resistance and potentially a reduced response magnitude compared to 30 °C. Considering the semiconductor nature of the MoS_2_, this general temperature dependence is expected [29].

The sensor relies on aptamers to selectively bind with cortisol, causing a conformational change that alters the electrical properties of the MoS_2_. The efficiency of aptamer-ligand binding is temperature-dependent [30]. Higher temperatures affect the binding kinetics, potentially reducing the stability of the aptamer-cortisol complex or altering the aptamer’s optimal conformation for binding. Specifically, elevated temperatures lead to increased thermal fluctuations that destabilize the hydrogen bonding and stacking interactions crucial for maintaining the aptamer’s specific three-dimensional structure, thereby reducing its affinity for cortisol or hindering the conformational change required for effective signal transduction. The binding affinity (measured by the dissociation constant) is inherently temperature-sensitive. Literature on cortisol-binding proteins, for example, shows a 16-fold drop in affinity as temperature rises from 35 °C to 42 °C. Similar trends are documented for aptamers, where higher temperatures favor the unbound state, leading to a smaller resistance change as fewer cortisol molecules successfully bind to the sensor surface [31,32]. This can explain the decreased sensitivity or a smaller resistance change upon cortisol exposure at higher temperatures, as behaved with the slightly lower response magnitude at 40 °C.

Table 2 compares the typical characteristics of cortisol sensors working at different operational principles, highlighting the advantages of the developed structures. While other platforms, such as electrochemical and optical methods, may offer competitive sensitivity or lower limits of detection, the current MoS_2_-based resistive sensor, particularly with its integrated ribs, demonstrates superior mechanical stability under continuous strain. This attribute is paramount for reliable performance in flexible, wearable applications where mechanical integrity directly impacts long-term sensing accuracy and consistency, offering a distinct advantage over more fragile analogues that may degrade under repetitive mechanical stress. It should be noted that the measurement units for the sensitivities are reported as in the original sources for transparency. They are not the same, because of the different implemented cortisol detection principles. Each method measures a different physical or chemical change that occurs in response to cortisol, such as electrical resistance, current, light intensity, or other transduced signals. However, the limit of detection, range, and response time parameters can be directly compared.

## 4. Conclusions

The designed chemoresistive sensors propose simpler designs compared to the complex microfluidic systems or intricate optical setups needed for some other methods. This results in lower manufacturing costs, enabling easier mass production of point-of-care devices and highlighting the potential of MoS_2_ for sensor applications. This study successfully investigated the effect of microresistor topology on the sensing characteristics of MoS_2_-based chemoresistive cortisol sensors, particularly focusing on their mechanical stability and performance under strain. Our findings demonstrate that the incorporation of ribs into the microresistor design significantly enhances mechanical stability, especially for the long-shoulder topology, by mitigating bending-induced degradation. Specifically, while the long-shoulder design with ribs maintained its functionality and provided clearer signals after bending compared to its ribless counterpart, the short-shoulder topology, even with ribs, proved less optimal, exhibiting greater sensitivity to mechanical noise and a reduced sensing performance.

Fluctuations in ambient or operational temperature (especially critical for wearable sensors interacting with human body temperature, ~36.7 °C) can introduce variability in readings. Without temperature compensation, the sensor’s accuracy in quantifying cortisol concentrations could be compromised, as a specific resistance reading might correspond to different cortisol levels depending on the temperature. The effect of temperature on the sensor’s response seems to be exacerbated after mechanical stress (bending), potentially indicating a more complex interplay between thermal and mechanical factors affecting the device’s integrity and function.

The designed chemoresistive sensors offer simpler designs compared to more complex microfluidic or optical setups, paving the way for lower manufacturing costs, easier mass production of point-of-care devices, and significantly contributing to the advancement of non-invasive hormone monitoring for improved public health outcomes.

## Figures and Tables

**Table 1:** Comparison of the different types of sensor designs for cortisol monitoring.

Feature	Planar	Micromachined	MoS_2_-Based
**Substrate type**	Rigid (glass, ceramic, Si)	Rigid (Si wafer)	Elastomeric polymer (PDMS)
**Stability goal**	Withstand shock and high temperature	Thermal stability and shock resilience	Flexibility, stretchability, and conformability
**Failure mechanism**	Brittle fracture under bending	Mechanical resonance structural fail under extreme force	Delamination, microcracking of the films under excessive strain or poor adhesion.

**Table 2:** Comparison of the obtained results for the favorable case (long-shoulder topology) with existing cortisol sensors.

Sensor Type	Recognition Element	Typical Concentration Range (e.g., ng/mL)	Sensitivity	Limit of Detection (LOD)	Response/Recovery Time	Other Characteristics Reported
**Electrochemical (Amperometric/Voltammetric)** [33]	Aptamers, Antibodies, MIPs	0.036 pg/mL to 5 µM (varies greatly)	7.9 kΩ/(pg/mL); 189.2 nA/µg/mL	As low as 0.36 pg/mL; ~0.214 nM	Rapid (often minutes or less)	High selectivity, good stability (MIP-based), suitable for miniaturization, low-cost.
**Optical (e.g., SPRi, Luminescent, Colorimetric)** [34]	Antibodies, Aptamers	0.2–8 ng/mL (SPRi); pm to nM range (Luminescent)	347.78°/RIU (SPR); 300-fold luminescent response	0.026 ng/mL (SPR); picomolar (luminescent)	Minutes	Often requires external reader (smartphone or instrument), high sensitivity, label-free (some SPR).
**Chemoresistive/FET-based** [35]	MIPs, Nanomaterials	0.01–20 ng/mL (FET); 10.874–173.981 µg/mL (Graphene)	Strongly varies by material and design	18 pg/mL (FET); 6.162 µg/mL (Graphene)	Rapid response time (seconds to minutes)	High sensitivity, label-free, robust materials, potential for direct detection.
**This work (long-shoulder topology)**	Aptamers	2.5–500 ng/mL (MoS_2_)	130.6 kΩ/ng/mL with ribs after bending; 0.9 kΩ/ng/mL without ribs after bending	2.5–500 ng/mL	1–2 min before bending; ~5 min after bending	High mechanical stability due to the ribs presence.

## Data Availability

The original contributions presented in this study are included in the article. Further inquiries can be directed to the corresponding author.
